# Language and economic behaviour: Future tense use causes less not more temporal discounting

**DOI:** 10.1371/journal.pone.0317422

**Published:** 2025-05-27

**Authors:** Cole Robertson, Seán G. Roberts, Asifa Majid, Robin I. M. Dunbar

**Affiliations:** 1 Centre for Language Studies, Radboud University, Nijmegen, The Netherlands; 2 School of English, Communication and Philosophy, Cardiff University, Cardiff, United Kingdom; 3 Department of Experimental Psychology, University of Oxford, Oxford, United Kingdom; University of Birmingham, UNITED KINGDOM OF GREAT BRITAIN AND NORTHERN IRELAND

## Abstract

Previous studies have found cross-cultural correlations between linguistic obligations for talking about future events and economic decisions like saving money. The hypothesis is that a grammatical obligation to use the future tense (e.g. *will*) causes speakers to perceive future rewards as temporally distal and therefore less valuable (“temporal discounting”). However, no studies have tested whether speakers actually temporally discount as a function of the extent to which they use the future tense. We present two studies which use a novel language-elicitation paradigm to do this, involving speakers of English (which obliges the future tense) and Dutch (which does not). We used mediation analysis to test how language-level differences in the grammatical obligation to use the future tense impact economic decisions *via* individual language use habits. However, we found that English speakers who habitually make greater use of the future tense actually discount less, not more. These results suggest obligatory future tense use is not responsible for previously-reported cross-cultural correlations. Instead, we suggest that a better explanation involves modal notions of certainty (the probability of an event occurring) rather than temporal distance (when an event will occur). Future tenses express high certainty, which makes the correct prediction that obligatory tense marking should cause less discounting. In contrast, the cross-cultural differences may be driven by variation in other aspects of future time reference, such as low-certainty modal terminology (e.g. *may*, *might*).

## Introduction

Linguistic relativity is the hypothesis that the peculiarities of the languages we speak can shape, augment, or otherwise alter our cognitive processes and thereby give rise to cognitive differences between populations [1]. While scholars continue to debate the depth and extent of linguistic penetration into cognitive processes [2,3], a growing body of evidence suggests that differences in online attentional demands during language use may grow into entrenched offline cognitive differences [4–6]. The hypothesis has recently gained popularity among economists, who report that cross-linguistic differences in the grammar of Future Time Reference (FTR [this refers to *any* linguistic utterance about the future.]) predict a range of behaviours which involve “intertemporal decision making”, where people must balance present versus future costs and rewards [7].

### The temporal hypothesis

Most research into this question has advanced a theoretical account we refer to as the temporal hypothesis [7]. This hypothesis is based on two observations. First, some languages oblige speakers to use the future tense for FTR, while others do not. For instance:

(1)
  - English
    *It will rain tomorrow.*
  - Dutch
    *Morgen*   *regent*   *het*.
    tomorrow   rain:PRS * it
    ‘Tomorrow it rains.’
    *The verb *regenen* ‘to rain’ is in the present tense (“PRS”), i.e. *regent* ‘It rains’. We use the Leipzig rules for interlinear glosses [8].

English speakers must use *will*, *be going to*, or *shall* in example (1a), whereas Dutch speakers are free to use the present tense (1b). Languages like English are referred to as “strong-FTR”, while languages like Dutch are referred to as “weak-FTR”. We refer to the distinction between weak- and strong-FTR as “FTR status.”

Specifically, weak-FTR languages are those languages which do not oblige the future tense in prediction-based contexts. Critically, example (1) is a *prediction*, and it is ungrammatical in English predictions to use the uninflected present tense, e.g. (*)*The Cardinals win on Sunday*. Weak-FTR languages are defined as those which permit such constructions for future *predictions*, and strong-FTR languages are any language which is not weak-FTR [7]. FTR strength is defined in terms of future predictions because it is common for languages, even strong-FTR languages, to permit the present tense in other common FTR contexts. The most important of these are *schedules* and *intentions* [9,10]. In fact, use of the present tense to refer to known future schedule is cross-linguistically universal, at least in European languages [10]. Taking such constructions into consideration might risk obviating any attempt at classifying language by FTR obligatorization. English is a good example: When referring to a schedule, the present tense is perfectly acceptable, e.g. *The Bears play at 7pm*. The same can be said of intentions, though in a more limited set of contexts, in particular near-term well-known future events, e.g. *I’m away next weekend*. As such, when we write of languages “obliging” the future tense, we follow Chen [7] in only referring to prediction-based FTR.

The second observation is that people tend to devalue (or “temporally discount”) outcomes as the time until they will occur grows longer [11,12]. For instance, given the choice of receiving $10 now or $10 in a month, most would choose the former because they would have temporally discounted the latter as a function of the delay. However, people have different time preferences: Give a choice between $10 now and $20 in a month and some will take $10 now, while others will choose to wait for $20 [13]. Temporal discounting is therefore usually measured by giving participants a series of binary choices like those above. Participants who temporal discount less tend to choose “future-oriented” options to wait for future rewards. Participants who discount more tend to choose “present-oriented” immediate rewards.

The temporal hypothesis predicts that weak-FTR speakers should temporally discount less than strong-FTR speakers, and therefore tend to be more future oriented. Two mechanisms are suggested to explain this [7]. Firstly, habitual use of the present tense for FTR in weak-FTR languages is suggested to “collapse” future time with present time, causing weak-FTR speakers to perceive future events as more temporally proximal than do strong-FTR speakers. The reasoning is that the present tense encodes notions of immediacy; for instance Chen [7] points out that it is a common literary practice to use the present tense to heighten the salience of past action. Conversely the future tense is suggested to encode notions of temporal distance, presumably because it is used to refer to temporally distal future events [7]. Obligatory use of the future tense is therefore hypothesized to cause speakers to construe future events are more subjectively distal than speakers of languages whose grammar permits the present tense to be used for FTR. This entails relatively increased discounting. We refer to this as the “distance mechanism” [7]. The other mechanism is that languages which have a future tense, “slice” time up into three segments– past, present, future— whereas languages which do not have a present tense only encode two segments— past, present + future. This is suggested to cause speakers of languages with a future tense to have more *precise* beliefs about the temporal location of future events, which would also lead to increased discounting [7]. We refer to this as the *precision* mechanism. Both mechanisms assume the future tense encodes temporal notions (distance, precision) and both predict that obligatory future tense use in strong-FTR languages will cause speakers to devalue future events (discount) relative to weak-FTR speakers. Together, we refer to these mechanisms as the temporal hypothesis.

### Empirical support for the temporal hypothesis

In the contribution which first proposed and tested the temporal hypothesis, [7] classed *N* = 129 languages in terms of FTR status. He then used FTR status to predict a set of behaviours which are plausibly downstream of temporal discounting. He found weak-FTR speakers were more likely to have saved money each year, retired with more assets, were less likely to have smoked, and more likely to practice safe sex. Additionally, they were healthier, as indexed by obesity, peak blood flow, grip strength, and physical exercise levels. Together, these findings indicate weak-FTR speakers prioritise future outcomes to a greater extent than strong-FTR speakers, suggesting they temporally discount less.

A number of studies have applied [7] approach to new outcomes. German (weak-FTR) speaking households were found to save more, smoke less, and resort to credit less than French (strong-FTR) speaking households in Switzerland [14]. Additionally, firms headquartered in weak-FTR-speaking countries engaged in more future-oriented behaviour [15], a pattern of results which has replicated across a variety of contexts [16–18]. Weak-FTR-speaking countries engaged in more future-oriented management of their economies [19,20]. Mandarin (weak-FTR) speakers had more pro-environmental attitudes than Korean speakers (strong-FTR) [21]; bilinguals expressed higher support for a fictitious pro-environmental “green tax” when they were tested in Estonian (weak-FTR) than Russian (strong-FTR) [22]; weak-FTR speakers (across a number of languages) were more likely to adopt environmentally responsible behaviours [23]; and weak-FTR-speaking countries enacted more pro-environmental policies and exhibited stronger commitment to global environmental cooperation [23]. Such decisions are compatible with an intertemporal interpretation, because climate change mitigation efforts tend to incur short-term costs but confer long-term benefits. In perhaps the strongest evidence to date, [24] assigned bilingual participants who spoke both a weak and strong-FTR language from 12 language pairs to complete an intertemporal choice task; they found speakers addressed in weak-FTR languages discounted less, as predicted by the temporal account, and that the variance in future-distance ratings was greater for weak-FTR speakers— the first empirical support for the precision mechanism.

### Problems with the temporal hypothesis

However, there are number of results which suggest more research is needed to understand the relationship between FTR grammaticisation in intertemporal decision making. Several studies have found future marking predicts pro-environmental behaviour (the opposite to the direction predicted by the temporal hypothesis). For instance, Germans given a pro-environmental choice task in the future tense (as compared with the present) were more likely to say they would invest money to plant trees [25]; across individual and national data, speakers of languages with inflectional FTR marking were more likely to perceive climate change as serious and support mitigation action [26]; and countries which spoke language with mandatory inflectional FTR marking had better climate change mitigation performance as measured by *CO*_2_ per capita, energy per capita, and energy per GPD [26]. These conflicting results suggest the temporal mechanisms proposed to relate FTR marking to future-orientation are not fully understood. This conclusion is supported by a study which found no support for the distance mechanism; in fact, weak-FTR speakers construed future events are more distal, the opposite to predicted direction [24]. This replicates our previous research, which found Dutch (weak-FTR) speakers rated future outcomes as more distal than English (strong-FTR) speakers [27, pp. 98–99]. This reverses the direction predicted by the temporal hypothesis. Taken together, these results suggest that the future tense does not encode temporal distance, cf. [7]. The other mechanism in the temporal hypothesis is that the future tense encodes temporal precision. This has found recent support, when variances were found to be greater when bilingual participants speaking weak-FTR language were asked to locate perceived distance using a slider [24]. However, if people speaking weak-FTR languages did represent future events less precisely, rewards marked by the future tense should be construed as less valuable, e.g. (“you get $10 in a week”) versus (“you *will* get $10 in a week”). However, no such pattern emerges. At least eight independent experiments have failed to find significant differences as a function of whether intertemporal choices were present tense or future tense [28–30]. This suggests the future tense choices are not construed as more temporally distal, or more temporally precise, since either mechanism would lead to increased discounting. Our previous research supports this conclusion: When participants used a slider to estimate the temporal distance encoded by a simple expression, no difference emerged as a function of whether the expression was framed in the future or present tense [, pp. 98–99]. Taken together these results suggest that the future tense does not encode notions involving temporal distance or precision, relative to present tense FTR, cf. [7].

If future tenses do not encode temporal distance relative to the present tense, what do they encode? In a previous study we investigated whether the future tense in English and Dutch encodes modal notions of high certainty [31]. Participants used a slider to rate future marked sentences between high and low-certainty. In both languages, participants rated future tenses as encoding high-certainty (%70-%80) [31]. This is perhaps un-surprising. Many contemporary scholars argue that *will* is not a tense at all, but rather part of English’s modal verb system [32–35]; similar arguments have been made about *zullen* [36]. Other approaches suggest *will*’s semantics are a mixture of temporal and modal [37,38]. While some emphasize *will*’s role in marking predictions as evidence it is a weak modal of low-certainty [35], others suggest it encodes modal strength (high-certainty) [33,39]. To understand these debates, it is necessary to expand the discussion beyond tenses strictly to the modal verb systems of which *will* and *zullen* are constituents. Paradigmatic analyses are a useful tool in this regard. Paradigmatic analyses attempt to understand the semantic contribution of a linguistic unit by contrasting it with the other units by which it may be replaced in a given context [40]. For instance, the future tense in English and Dutch can be formed with either a modal verb (English *will/shall*; Dutch *zullen* ‘will’) or a “go-based”, construction (English *be going to/gonna*; Dutch *gaan* ‘be going to’). These are constituents of FTR systems comprising other modals which— at least among those which are permitted in prediction-based FTR— encode modal weakening relative to the future tense markers (English *could, may, might, should, would*; Dutch *kunnen* ‘may’) [41]. This modal weakening was present in our previous study: When participants rated the certainty encoded by modally weak verbs like *may*, it was approximately %30– %40 [31]. A paradigmatic analysis therefore suggests that *will* and *zullen* encode high-certainty notions relative to the other modals, e.g. *De beren zullen vanavond winnen* ‘The Bears will win tonight’ expresses high certainty relative to *De Beren kunnen vanavond winnen* ‘The Bears might win tonight’. This interpretation was supported by our previous study [31]. For these reasons we conclude that a plausible interpretation of FTR semantics is that future tenses encode high-certainty FTR.

### Hypotheses

We hypothesize that obligatory use of the future tense would cause strong-FTR speakers to construe future outcomes as more certain (less risky). We further hypothesise this would cause them to discount less not more. cf. [7]. Recent accounts treat modal expressions as scalar operators which map transparently onto notions of probability. Rather than Boolean quantification over possible worlds, modal semantics involve encoding the likelihood of events on a one-dimensional scale between high (*p* = 1) and low (*p* = .5) certainty [42–44] (*p* = .5 for low-certainty because *p* = 0 indicates high certainty in the non-occurrence of some event). Evidence suggests scalar accounts capture modal semantics better than notions of Boolean quantification, since the latter yields incorrect predictions in some linguistic contexts [42]. Thus, future tenses plausibly encode high certainty, this maps onto probability, and people discount as a function of probability as well as time [13,45,46]. Imagine someone has a belief *F* about some future probability *p*. The obligation to use high-certainty future tenses would lead to high-probability construals of future events in strong-FTR speakers $F_{S}(p_{FUT})>F_{W}(p_{FUT})$, where $p_{FUT}$ indicates we are strictly referring to *future* probabilities. Since people discount as a function of probability as well as time [13,45,46], this would lead directly to decreased temporal discounting.

This means Chen’s (2013) temporal account and our modal account have different predictions about how obligatory future tense use should impact temporal discounting. Specifically, we argue that the future tense encodes high-certainty and that therefore strong-FTR speakers will construe future events in high-certainty terms (i.e. $F_{S}(p_{FUT})>F_{W}(p_{FUT})$) and that this will lead to decreased discounting. We refer to this as the “high-certainty” hypothesis. Conversely, [7] argues that the future tense encodes temporal distance or temporal precision. In the first case, strong-FTR speakers construe future events as more distant (i.e. $F_{S}(t_{d})>F_{W}(t_{d})$), in the second, strong-FTR speakers construe future events with more temporal precision (i.e. $F_{S}(t_{p})>F_{W}(t_{p})$). Either of these differences in belief lead to *increased* discounting [, conditional on some assumptions]. Resolving which of these is the case is an empirical question: Does increased use of the future tense in strong-FTR languages lead to increased discounting [7] or does it lead to decreased discounting? Studies 1 & 2 attempt to answer this.

## Study 1

Our goal in Study 1 was to ascertain whether increased future tense use in English caused higher or lower temporal discounting. Higher discounting supports the temporal account of tense semantics (distance/precision). Lower discounting supports the modal account (high certainty).

### Methods

#### Participants.

We conducted a power analyses to estimate necessary sample size. There being no clear precedent in the literature to form the basis of a statistical power analysis, we used a rule of thumb methodology to estimate necessary power. Green [47] recommends the following formula for estimating statistical relationships: *N*>50 + 8*m*, where *m* is the number of independent variables, for us 8, counting demographic covariates. Since the interaction in the reported mediation models mean we need to estimate the relationship between discounting and future tense use independently in each language, we multiply this by 2: (50+8*8)*2=N>228.

An initial sample of *N* = 240 adult participants completed the study. However, one participant was excluded because their answers to the FTR-elicitation task were given in bad-faith and four were excluded because of missing demographic data. This left a final sample of *n* = 235 (*n* = 113 in English [*n* = 62 females, *n* = 49 males, *n* = 2 other], and *n* = 122 in Dutch [*n* = 106 females, *n* = 16 males]). Given potential for sex differences in risk preferences, and differences in sex composition between the English and Dutch samples, sex, as well as all demographic variables were included as covariates in all reported analyses in Study 1 and Study 2. Doing so did not substantively change any reported model parameters or posterior probability estimates (SI 1). English speakers were recruited from the University of Oxford and from Prolific Academic, and Dutch speakers were recruited from the Max Planck Institute for Psycholinguistics (MPI). All participants were screened such that they spoke the respective language of the survey as their first language, and were at least 18 years old. Data were collected between 16 January and 3 February, 2017. Prior to participating, all participants provided informed consent using an online form. Ethical approval for the study was granted by the University of Oxford internal review board, ref. no. R39324/RE001, and by the MPI for the data collection that occurred there. All participants were remunerated.

#### Materials.

Study 1 comprised two tasks: an FTR-elicitation task designed to establish individual future-referring language and an intertemporal-choice task designed to measure temporal discounting.

**The FTR-elicitation task:**

To establish FTR language we created a new FTR-elicitation task which we based on the EUROTYP FTR questionnaire [9,10], on which FTR status is predominantly based [7]. In this task, participants were given a context and a target sentence. The main verb in the target sentence was uninflected, and participants were asked to render the target sentence given the context. All contexts referred to future events. Before starting the FTR-elicitation task, participants were given three past-time examples. Participants were advised that there “were no correct answers,” and that they should complete the questionnaire sentences, “as though they were speaking to a close friend.” See SI 1 for items.

We were concerned that the grammatical constraints of FTR-elicitation items did not overwhelmingly drive results. In other words, we reasoned there might be certain items which did not permit any variation in possible responses. This would mean that speaker-level differences in FTR usage could not influence elicited FTR data. This would not have been ideal since we wanted to correlate speaker-level elicited FTR language with temporal discounting. We therefore conducted a pilot study with the full (48 item) FTR-questionnaire and used these data to choose a subset of items. Data for the pilot were collected between July and October, 2016, from the MPI participant panel (Dutch and German), and Oxford University students (English). Final *N* = 64 (*n* = 45 English and *n* = 19 Dutch). Ethical approval for the pilot was granted by the University of Oxford internal review board, ref. no. R39324/RE001, and by the MPI for the data collection that occurred there. All participants were remunerated. To choose items, using the pilot data, we regressed future tense use over an intercept allowed to randomly vary by both item and participant. We selected those items for which “item effects” explained the least variance. In other words, these were the language-matched sample of questions for which answers were least constrained by context (see SI 1). This process resulted in a sample of *N* = 29 items, which were the items participants saw in Study 1. However, three items were problematic for two reasons. Firstly, inclusion of tense or modal words in the contexts (*go, think*, etc.) made it impossible to identify whether participants had used the words themselves or had copied the prompt. Secondly, for one item, it was impossible to tell whether participants interpreted it as referring to present or future time. The item was: “[Q: Do you think your dad will go to sleep?] A: Yes, he {BE} tired.”. Participants overwhelmingly responded with e.g. *Yes he is tired*. It is impossible to tell whether this means that he is tired *now* or that he will be tired *later*. Data from these problematic items were therefore excluded from analyses.

Stimuli in the FTR-elicitation task varied according to 3 within-subjects factors: (1) distance from present (later today, tomorrow, one week, six months, one year, two years, ten years, and 25+ years, indeterminate, and ongoing predictions; (2) modality condition (high-certainty, low-certainty, neutral); and (3) FTR-mode (whether the context involved a prediction or an intention). (After exclusions, no schedule-based FTR items remained.)

Following Dahl [10], we defined predictions as contexts in which the future outcome is not under the control of the speaker, requiring the speakers to estimate the likelihood of the event, e.g. “It {RAIN} tomorrow.” We defined intentions as contexts which generally are under the control of the speaker (when people know what they or others intend to do), e.g. “I/you {DRIVE} to the store.” Temporal distance indexed the temporal distance from the time of speech. We operationalised temporal distance by using temporal adverbials in the context (“one month”, “two weeks”, etc.) or by referring to well-known events which would occur a specific length of time after data collection, e.g. the U.S. election. We operationalised modality condition using parenthetical verbal “certainty information”, which indicated how certain the participant was supposed to be:

**Table:** 

neutral	**Context**: It’s no use trying to swim in the lake tomorrow...
	**Target**: ...The water {BE} cold (then).
low-certainty	**Context**: Q: What is the weather forecast for next week?
	**Target**: A: It {RAIN} (a 50% chance).
high-certainty	**Context**: Don’t bother investing in the tech industry...
	**Target**: ...Silicon Valley {CRASH} within two years (I’m very sure).

In the high-certainty condition, participants were told to imagine they were highly certain, in the low-certainty condition they were told to imagine they were highly uncertain, and in the neutral condition they were not given any certainty information. To avoid participants simply reproducing the certainty information in their responses, above each item was the directive to “omit material within parentheses.”

Free-text responses were classified using the FTR-type classifier [48], which is a deterministic closed-vocabulary keyword-based classification program we wrote in Python [49]. Simply scoring responses which were formally future tensed as “future tense” would have resulted in confounded results because it is possible in English and Dutch to encode modal notions in addition to using either the future or present tense, e.g. *It will possibly rain tomorrow* (future + modal), *Rain tomorrow is possible* (present + modal). We reasoned that the notion which is being marked semantically is the critical determinant of cross-linguistic effects on cognition, e.g. relativity effects emerge when language points attention to features of the world (i.e. semantics) and these features become engrained over time until they are entrenched as offline cognitive patterns. The critical thing for the FTR-classifier to encode is therefore which semantic notions are encoded by FTR statements. With that in mind, it is important to notice that the semantic contribution of the future tense is drastically different when other modal markers are present. In *It will rain tomorrow*, the future tense (*will*) arguably encodes high-certainty (in contrast to the other modals, e.g. *may, might*). Conversely, in *It will possibly rain tomorrow*, it does not. The low-certainty semantics of *possibly* “dominate” *will*. We built the FTR-type classifier to reflect this linguistic fact so that FTR statements were only counted as “future” or “present” when no other modal markers were used. We implemented the following class schema.

Future tense: Responses were classed as future tense if they used commonly accepted “future” auxiliaries or explicit temporal adverbials (English *will, shall, be going to, about to*; Dutch *zullen* ‘will’, *gaan* ‘be going to’, *staat op* ‘about to’). Any response exhibiting these words, without additional modal words (high or low-certainty, e.g. English *might, may, possibly, definitely*; Dutch *kunnen* ‘may’, *mogelijk* ‘possibly’ *zeker* ‘certainly’) was counted as future tense. Responses which met these criteria were scored with (future tense = 1), otherwise (0).

Present tense: Responses were classed as present tense if they conjugated the main verb in the target sentence using the present tense, were not classed as future tense, and did not use additional modal words (as for the future tense). Responses which met these criteria were scored with (present tense = 1), otherwise (0).

The FTR-type classifier’s predictions using these schema were verified against a test set of *N* = 926 (*n* = 420 English, *n* = 506 Dutch) responses from Studies 1 and 2, which were classified by trained human raters, and all accuracy metrics were >.96 (SI 1).

In order to compare elicited FTR language with temporal discounting at the participant-level, we used elicited FTR language means per participant as our dependent variables. These were equal to $FTR_{j}=\sum_{n}^{i=1}\frac{FTR_{ij}}{n_{j}}$, where *FTR* responses are scored for participant *j*, and *n* is the total number of responses by that participant. We calculated this metric independently for the present and future tense. In other words, these metrics equalled the proportion a participant used the future tense *FUT*_*j*_ and present tense *PRES*_*j*_ out of the total elicited utterances.

**The intertemporal-choice task:**

In this task, participants made a series of binary choices between a small reward given now (the Smaller-Sooner Reward [SSR]), and a larger reward given after a delay (the Larger-Later Reward [LLR]). For example, “Would you rather have £7.50 now or £10 in two months?” Dutch participants were given amounts in euros. The amount of the larger-later reward was always £10/€10. Prior to starting the temporal discounting task participants were told to “try to answer quickly and honestly, without thinking about it too much” and completed a training example with a larger-later reward of £10,000. The amounts of the smaller-sooner reward were £4.00-£9.50 by increments of £0.50; the delays of the larger-later reward were one day, two days, one week, two weeks, one month, six months, and two years. Amounts and delays were fully crossed to produce a battery of test items ($12_{amounts}\;\times \;7_{delays}=84_{items}$). Participants were told that the amounts in the intertemporal choice task were hypothetical, and they would be remunerated a fixed amount regardless of their answers.

To calculate participant-level discounting we followed the procedure given in [50]. Research has shown that the following hyperbolic function fits real and hypothetical delay-discounted value well [50,51]:

$$V=\frac{A}{1+kD}$$ (1)

where *V* is the subjective value of a delayed reward *A* at a given delay *D*, and *k* is a scaling parameter which captures individual differences in discounting. Higher values of *k* imply more discounting, i.e. lower subjective valuation of the delayed larger-later reward, *V*.

To derive participant-level *k*, we followed [50]. This involved calculating hypothetical values of *k* and retaining for each participant some *k* which best predicted empirical choices. Specifically, we calculated all *k*_1−*n*_ at indifference between LLR and SSR, i.e. all *k*s such that $SSR=LLR(1+k_{i}D)$ for all *n* = 84 values of *SSR* by *D* under the study. We then created hypothetical intertemporal choices for each *k*_1−*n*_ by using eq. 1, i.e. if $SSR>\frac{LLR}{\left(1+kD\right)}$, we predicted *choice* = *SSR*, otherwise *choice* = *LLR*. We then retained *k*_*i*_ for each participant *p*_*j*_ which had the highest proportion of matches against *p*_*j*_ empirical choices. When more than one *k* had an equal number of matches, we took the geometric mean of all matching values of *k* [50]. This method accurately captured participant-level discounting, correctly predicting 93.04% of all intertemporal choices. Since *k* tends to be approximately exponentially distributed, we used *log*_*e*_(*k*) as our dependent variable in order to better approximate regression assumptions about error normality [50].

#### Procedure.

The survey was hosted on Qualtrics, and all participants completed it online. English first-language speaking participants completed the survey in English, and Dutch first-language speaking participants completed the survey in Dutch. Before commencing, participants were asked to confirm their first language matched the survey language. If they did not, they were immediately ejected from the survey. Participants then answered some demographic questions (age, sex, income, education, marital status, and employment status). We never found that any of these significantly impacted outcomes, so they are henceforth disregarded (SI 1). All participants then completed the FTR-elicitation task and then the intertemporal-choice task. In both tasks, item order was fully randomised and one item was displayed per page.

### Results

English speakers used more future and fewer present tense constructions, Table 1, which supports the classification of English as strong- and Dutch as weak-FTR. Future tense use was negatively correlated with discounting in English but not Dutch, Table 2. This suggests the English future tense encodes high certainty rather than temporal distance.

**Table 1:** Descriptive statistics for Studies 1 and 2.

study	variable	language	$\bar{X}$	SD	$\tilde{X}$	*MAD* *	min	max	*Q*_0.25_ ^†^	*Q*_0.75_ ^†^
Study 1	future tense	English	0.59	0.13	0.62	0.11	0.19	0.85	0.52	0.69
		Dutch	0.33	0.16	0.31	0.14	0.00	0.68	0.23	0.42
	present tense	English	0.06	0.08	0.04	0.06	0.00	0.42	0.00	0.08
		Dutch	0.40	0.18	0.38	0.13	0.04	1.00	0.31	0.5
	*log*_*e*_(*k*)	English	-3.15	2.11	-2.87	2.28	-7.20	1.54	-4.90	-1.80
		Dutch	-3.17	1.92	-2.89	2.00	-7.20	0.13	-4.57	-1.64
Study 2	future tense	English	0.59	0.16	0.62	0.12	0.04	0.92	0.5	0.7
		Dutch	0.20	0.18	0.17	0.19	0.00	0.88	0.04	0.29
	present tense	English	0.07	0.09	0.04	0.06	0.00	0.75	0.00	0.12
		Dutch	0.55	0.24	0.54	0.25	0.00	1.00	0.38	0.75
	*log*_*e*_(*k*)	English	-2.97	1.32	-2.53	1.09	-5.49	-1.79	-3.82	-1.79
		Dutch	-3.77	1.38	-3.64	2.09	-5.49	-1.79	-5.49	-2.67

^*^Median absolute deviation.

^†^Quartiles.

**Table 2:** Pearson correlations for variables in Studies 1 and 2, by language

study	language	variable	future tense	present tense
Study 1	English	future tense		
		present tense	-0.57***	
		*log*_*e*_(*k*)	-0.20*	0.09
	Dutch	future tense		
		present tense	-0.83***	
		*log*_*e*_(*k*)	0.10	-0.10
Study 2	English	future tense		
		present tense	-0.59***	
		*log*_*e*_(*k*)	-0.15**	0.12*
	Dutch	future tense		
		present tense	-0.73***	
		*log*_*e*_(*k*)	-0.04	0.01

^***^p<.001; ^**^p<.01; ^*^p<.05; ·p<.1

#### Mediation analysis.

**Fig 1:**
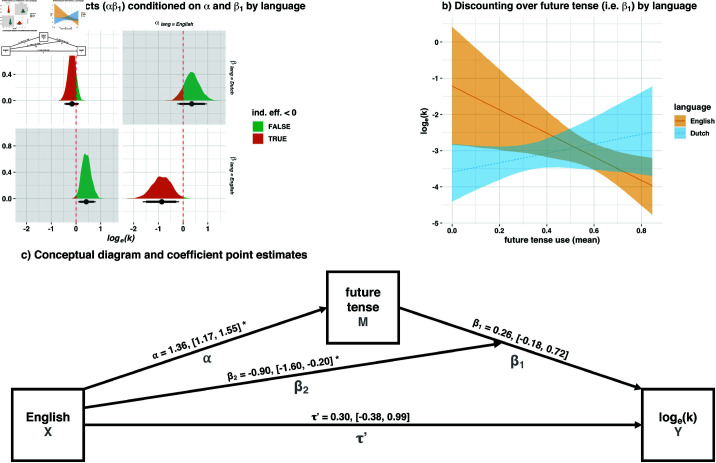
Study 1 mediation analysis, with conditional indirect effects of language on temporal discounting via future tense use. Credibility intervals are plotted at 90% (thick bars) and 95% (thinner whiskers). For two-tailed hypotheses, thin bars represent cut-off criteria; for one-tailed hypotheses, thick bars do (providing sign is as predicted). Counterfactual parameters are in grey. b) Marginal effects of participant-level *log*_*e*_(*k*) values over mean future tense use conditioned on language (i.e. β for each language) with 95% credibility intervals. Lower values indicate less discounting. c) Conceptual model diagram; interaction term is represented by paths intersecting. Full parameters are given in Table 3, keyed by path name. To ease interpretation, figures throughout use raw predictor values, not the mean-centred *z*-scaled variables used in regressions. ^*^95% *CI* does not contain zero.

Our hypothesis is mediational; English grammar is hypothesised to mandate more future tense marking relative to Dutch, and future tense marking in English is hypothesised to cause high-certainty representations of future events in English speakers and therefore less discounting. Put another way, individual English language use *mediates* a relationship between English grammatical obligations and temporal discounting. To test this, we conducted a mediation analysis. In mediation analyses, the predictor is referred to as *X*, the mediator is referred to as *M*, and the outcome is referred to as *Y* (see Fig 1). The X→M path is referred to as α, the M→Y path is referred to as β, and the X→Y path is referred to as $\tau^{\prime }$, or the “direct effect”. The X→M→Y path is referred to as the “indirect effect”; it can be estimated as the product of the paths involved (αβ). It captures the effect of *X* on *Y* via *M* while controlling for the direct effect ($\tau^{\prime }$). The direct effect captures the effect of *X* on *Y* while controlling for the indirect effect (αβ). The “total effect” is the sum of αβ and $\tau^{\prime }$, and captures the total effect of *X* on *Y*, as in a normal regression [52]. To allow our model to capture language-wise differences in temporal discounting over future tense, we allowed β to be conditional on language by including an interaction term for future tense by language, see Fig 1.

**Table 3:** Regression coefficients for mediation analyses Studies 1 and 2.

study	path name	outcome	predictor	estimate	*SE*	low-95%*CI*	high-95%CI**	
Study 1	$\lambda_{1}\;(\alpha_{lang=dut})$	future tense	Intercept	-0.65	0.07	-0.78	-0.52	*
	$\lambda_{2}$	*log*_*e*_(*k*)	Intercept	-3.00	0.24	-3.46	-2.52	*
	$\alpha \;(\alpha_{lang=eng})$	future tense	English	1.36	0.10	1.17	1.55	*
	$\beta_{1}$	*log*_*e*_(*k*)	future tense	0.26	0.23	-0.18	0.72	
	$\tau^{\prime }$	*log*_*e*_(*k*)	English	0.30	0.35	-0.37	0.99	
	$\beta_{2}$	*log*_*e*_(*k*)	future tense * English	-0.90	0.36	-1.60	-0.20	*
Study 2	$\lambda_{1}\;(\alpha_{lang=dut})$	future tense	Intercept	-0.75	0.04	-0.82	-0.67	*
	$\lambda_{2}$	*log*_*e*_(*k*)	Intercept	-3.82	0.12	-4.04	-3.6	*
	$\alpha \;(\alpha_{lang=eng})$	future tense	English	1.51	0.05	1.40	1.62	*
	$\beta_{1}$	*log*_*e*_(*k*)	future tense	-0.07	0.11	-0.29	0.15	
	$\tau^{\prime }$	*log*_*e*_(*k*)	English	1.10	0.17	0.77	1.44	*
	$\beta_{2}$	*log*_*e*_(*k*)	future tense * English	-0.26	0.17	-0.59	0.07	

All predictors were mean-centered and z-scaled so coefficient point estimates represent predicted changes in outcomes as a function of changes in 1 standard deviation in the predictors. Point estimates are the mean of the posterior probability distribution. Bayesian *R*^2^ equivalents are reported in the main text.

*Bayesian 95% Credibility Interval (CI) does not contain zero.

**Credibility intervals are reported at 90% in the main text because they test one-tailed hypotheses.

We used *log*_*e*_(*k*) as our outcome variable (*Y*), language (English = 1, Dutch = 0) as our predictor (*X*), and participant-level mean future tense use as our mediator (*M*). The model therefore took the following form:

$$fut_{i}=\;\lambda_{1}+\alpha lang_{i}+e_{1i}\;log_{e}(k{)}_{i}=\;\lambda_{2}+\tau^{\prime }lang_{i}+\beta_{1}fut_{i}+\beta_{2}fut_{i}lang_{i}+e_{2i}$$ (2)

where $\lambda_{1,2}$ are intercepts, and α, $\tau^{\prime }$, and $\beta_{1}$ are slope coefficients as described. Because $\beta_{1}$ is moderated by language, the indirect effect is calculated conditional on $\beta_{2}$, i.e. $ind.eff.=\alpha (\beta_{1}\;+\;\beta_{2}language)$. For Dutch (*language* = 0), this simplifies to the normal indirect effect as described, but for English (*language* = 1) the interaction term $\beta_{2}$ is added. For parity, we report the indirect effect conditioned on both α and β by language, where $\alpha_{eng}$ is α, and $\alpha_{dut}$ is the future tense intercept $\lambda_{1}$. This allows us to estimate the effect of observed English and Dutch levels of future tense use conditional on observed English and Dutch relationships between future tense use and discounting, see Fig 1.

Bayesian statistics are well-suited to mediation analyses. As well as making no assumptions about the normality of sampling statistics, they allow for straight-forward inferences about any transformation of model parameters (i.e. path products) through carrying out the desired operation on posterior probability distributions [53]. We therefore used the *brms* package [54] in *R* [55] to estimate model parameters using a no U-Turn Hamiltonian Monte Carlo sampling procedure [56,57] with uninformative priors (i.e. Unif(−∞–∞)), 4 chains, 2,000 iterations, and a burn in discard of 1,000 (the defaults). Inspection of caterpillar plots and $\hat{R}$s indicated that the estimation procedure had converged [53].

The predictors explained a large proportion of future tense variance and a small proportion of discounting variance, $R_{future}^{2}=0.46,\;CI_{95\%}=[0.38,0.52]$, $R_{log_{e}(k)}^{2}=0.04,\;\;CI_{95\%}=[0.01,0.09]$. These are a Bayesian adaptation of *R*^2^, including spread statistics for values at the 2.5% and 97.5% percentiles [58].

Fig 1a reports indirect effects conditioned on language-wise differences in α and β, as explained. Counterfactual thinking is necessary to understand these parameters [59]. For instance, Fig 1a, *bottom left* reports the counterfactual effect of the observed Dutch levels of future tense use (α|lang=Dutch) combined with the observed English relationship between future tense use and discounting (β|lang=English). The significant effect indicates that low use of the future tense in Dutch would cause increased discounting, were discounting over future tense use the same in Dutch as in English (which it is not).

The quantities of interest are the non-counterfactual parameter estimates. In particular, we are interested in the (true) effect of English use of the future tense, i.e. the effect given $\alpha_{eng}\;\&\;\beta_{eng}$. This effect is negative, Fig 1a*, bottom right*, and significant, $Est.=-0.87,\;\;CI_{90\%}=[-1.5,-0.28],\;\;pp=\mathrm{.991}$. In these parameter summaries, *Est*. is the mean of the posterior probability distribution, CI is the x% credibility interval (90% in this case because the hypothesis is one-tailed, i.e. the high-certainty hypothesis αβ<0), and “*pp*” reports the probability that the posterior estimate of the effect matches the direction of the hypothesis. In contrast to frequentist *p* values, *pp*>.95 indicates evidence that the parameter matched the prediction exceeds 95% [54]. In summary, English speakers used significantly more future tense constructions than Dutch speakers, i.e. α in Fig 1c. In English, the relationship between future tense use and discounting was negative, Fig 1b. As a consequence, the indirect effect was negative (as the modal account predicts), not positive (as the temporal account predicts), i.e. English speakers used more future tense constructions, and this drove them to discount *less*, makes sense if *will* encodes high certainty (modal account) but not if it encodes temporal distance (temporal account).

### Discussion

The principal result of Study 1 is that obligatory future tense use in our strong-FTR language (English) resulted in less rather than more discounting. This suggests the English future tense encodes high certainty, not temporal distance or precision, cf. [7]. This supports the high-certainty and not the temporal account.

## Study 2

The Study 1 results undermine the widely-cited temporal account which has been used to motivate and interpret a great deal of work [7,14–22,30,60–67]. Rather than high future tense use in English resulting in more temporal discounting, it resulted in less. *If* our findings generalise to other languages— a question requiring more research— this would mean that something other than obligatory future tenses is driving observed effects of FTR status on intertemporal behaviour. Given the extent of the findings this result undermines, a replication seemed appropriate. In Study 2, we therefore replicated Study 1, but with a larger sample. We also adjusted the amounts and delays in the intertemporal-choice task; our aim was to increase the resolution of the task to better capture correlations between future tense use and discounting (SI 1).

### Methods

#### Participants.

An initial sample of *N* = 625 participants completed Study 2 (this number was determined by a power analysis, SI 1). However, *n* = 7 participants were excluded because inspection indicated their FTR-elicitation responses were given in bad faith and *n* = 12 participants were excluded because a survey error resulted in excessive missing data. After these exclusions, a final sample of *n* = 606 participants completed Study 2 (*n* = 301 in English [*n* = 134 males, *n* = 163 females, and *n* = 4 other], and *n* = 305 in Dutch [*n* = 127 males, *n* = 177 females, and *n* = 1 other]). English speakers were recruited from Prolific Academic and Dutch speakers from Qualtrics. All participants were screened such that they spoke the respective language of the survey as their first language, and were 18 or over. Data were collected between 4 October 2018 and 1 November 2018 (Dutch) and on 28 May 2020 (English). Ethical approval for the study was granted by the University of Oxford internal review board, ref. no. R39324/RE001. Prior to participating, all participants provided informed consent using an online form. All participants were remunerated equally.

#### Materials.

Study 2 again comprised two tasks: the same FTR-elicitation task as in Study 1, and an intertemporal-choice task with updated amounts and temporal delays.

**The FTR-elicitation task:**

The FTR-elicitation task was identical to Study 1, and the dependent variables were the same. However, in addition to the three items which needed to be excluded from Study 1, an additional two items were excluded from Study 2, leaving a final sample of *N* = 24 items. This was because an oversight meant that a specific temporal adverb (“by 2018”) was in the past at the time of data collection (SI 1).

**The intertemporal-choice task:**

The amounts of the smaller-sooner rewards in Study 2 were £7.50-£9.25 by increments of £0.25, and the delays of the larger-later reward were 2 weeks, 1 month, 1½ months, 2 months, 2½ months, 3 months, 4 months, and 4½ months. (Three and a half months was accidentally not included due to an oversight.) Amounts and delays were fully crossed to produce a battery of test items ($8_{amounts}\;\times \;8_{delays}=64_{items}$). The dependent variable was again *log*_*e*_(*k*), as in Study 1. The procedure for estimating participant-level *k* again accurately captured participant-level discounting, correctly predicting 94.29% of responses.

#### Procedure.

Procedure was identical to Study 1. English-speaking participants completed the survey in English and Dutch-speaking participants completed the survey in Dutch. Task order, presentation, and demographic covariates were exactly replicated.

### Results

Replicating Study 2, future tense use was negatively correlated with discounting in English but not in Dutch, Table 2. This suggests that higher future tense use in English would again result in less discounting, as the modal hypothesis predicts.

#### Mediation analysis.

To test this, we re-estimated the same model with the same estimation parameters as in Study 1 eq. 2. This time, the model explained a similar proportion of future tense use variance and a slightly higher proportion of discounting variance, $R_{future}^{2}=0.57,\;\;CI_{95\%}=[0.53,0.6]$, $R_{log_{e}(k)}^{2}=0.09,\;\;CI_{95\%}=[0.05,0.14]$.

**Fig 2:**
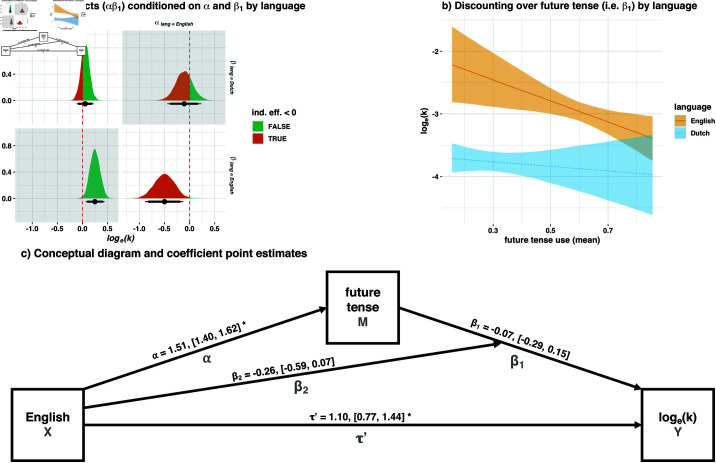
Study 2 mediation analysis, with conditional indirect effects of language on temporal discounting via future tense use, moderated by language. (**a**) Estimates of conditional indirect effects; see Fig 1 for notes on interpretation. (**b**) Marginal effects of participant-level *log*_*e*_(*k*) over mean future tense use conditioned on language (95% credibility intervals). Lower values indicate less discounting, so higher estimations of delayed future rewards. (**c**) Conceptual model diagram; full parameters are given in Table 3, keyed by path name. Study 1 replicated: The indirect effect in English was negative and did not contain zero (*a, bottom right*). The indirect effect in Dutch was non-significant (*a, top left*). ^*^95% *CI* does not contain zero.

Once again, we found that discounting over future tense use was negative in English (Fig 2b) and that the indirect effect conditioned on English was negative (Fig 2a*, bottom right*), as the high-certainty hypothesis predicts, $Est.=-0.5,\;\;CI_{90\%}=[-0.83,-0.19],\;\;pp=\mathrm{.995}$. This replicated our main Study 1 finding that higher future tense use in English resulted in decreased— rather than increased— discounting.

Again, discounting over future tense use in Dutch was not significant, i.e. $\beta_{1}$ for Study 2 in Table 3 (see Fig 2b). Again, the indirect effect conditioned on Dutch was not significant, $Est.=0.05,\;\;CI_{90\%}=[-0.08,0.19],\;\;pp=\mathrm{.252}$ (Fig 2a*, top left*).

### Discussion

In Study 2— as in Study 1— higher use of the future tense in English resulted in less, not more, temporal discounting. This suggests that higher English future tense use is not causally implicated in higher English discounting, as is hypothesised by the temporal account. This entails that other factors are responsible. A plausible account is that time preferences are affected by constraints in English that oblige low-certainty modal FTR— e.g. *could, may*, or *might*. We address this idea below.

## General discussion

Most contemporary research into FTR grammaticisation and temporal discounting has been theoretically framed using the temporal account proposed in [7]. According to this account, the future tense encodes either temporal distance or precision relative to the present tense. Either of these mechanisms are hypothesised to cause strong-FTR speakers to temporally discount more. In contrast, we gave an account that future tenses encode high-certainty modality. We hypothesised that obligatory future tense use in strong-FTR languages would therefore cause speakers to discount less. We referred to this as the high-certainty hypothesis.

The results are clear: It is difficult to construe a way in which they support the temporal hypothesis. Most importantly, we found that future tense use was negatively related to discounting in English, and the effect of future tense use in English resulted in decreased discounting— the direction predicted by the high-certainty hypothesis. This effect replicated in Study 2, using a larger independent sample. In so far as it may generalise, this undermines the temporal hypothesis that obligatory future tense use in strong-FTR languages is driving observed increased discounting. This suggests that much of the work interpreted to support the temporal hypothesis should be reconsidered in this light.

Intriguingly, we found no effect of future tense use on temporal discounting in Dutch. This suggests that the semantics of the future tense work differently in Dutch than in English. A paradigmatic analysis may be useful. In English, the future tense is the highest-certainty construction type which is permitted for prediction-based FTR; *will, be going to*, and *shall* therefore contrast with a set of low-certainty modal verbs (*could, may*, etc.). These paradigmatic contrasts suggest that the English future tense encodes high certainty. In Dutch, the present tense is possible: *Zullen* and *gaan* may be paradigmatically contrasted with present tense FTR, e.g. *Morgen regent het*. ‘Tomorrow it rains’. Relative to such unmarked statements of fact, any modalisation may be weaker. This suggests the Dutch future tense encodes modal weakening, or lower certainty. The paradigmatic oppositions of future tenses may therefore differ as a function of the cross-linguistic differences indexed by FTR status. This could explain null effects of future tense use on discounting in Dutch. Such findings undermine the validity of continuing to use FTR status as a descriptor of cross-linguistic variation in FTR grammaticisation, i.e. since FTR status *de facto* assumes the obligatory FTR marking has the same effect in all *N* = 129 indexed languages. These results point research toward future investigations involving fewer, more closely studied, languages; such research would afford language-specific treatment of the FTR grammars involved and any corollary effects on discounting.

### What is responsible for observed discounting in strong-FTR speakers?

To the extent they may generalise, the negative indirect effects of future tense use undermines the temporal mechanisms hypothesised in [7]. What is to be made of findings that FTR status is a consistent predictor of intertemporal behaviour [7,14–22,30,60–67]? On the one hand, correlational research involving linguistic predictors is notorious for producing spurious relationships due to non-independence between languages resulting from historical processes of cultural inheritance which see causally unrelated bundles of traits being passed on from antecedent to descendant cultures [68]. When linguistic history controls are added, results have been mixed [69], or have failed to replicate [63]. Reported correlations may be spurious. On the other hand, FTR grammaticisation could be impacting temporal discounting, just not via the tense-based temporal mechanisms hypothesised in Chen [7]. In this case, new theoretical accounts would need to be developed to explain the observed effects of FTR status.

One possibility is that strong-FTR languages oblige the use of low-certainty modal constructions for FTR. English is a good example. When English speakers make predictions, it is not obligatory to use the future tense, cf. [7]. What *is* obligatory is a modal verb. For instance, in example (1), it is acceptable to say any of *It can/could/may/might/shall/should/will/would/is going to rain tomorrow*. With the exception of the future tense constructions (*shall, be going to*), the other modals generally encode modally weak notions of low certainty. In a previous study using a similar FTR-elicitation task, we found English speakers used more low-certainty FTR constructions than Dutch speakers [, pp. 86–95]. This indicates that in English strong-FTR grammar obliges speakers to use low-certainty modals to talk about the future. Could this cause them to construe future events as more risky?

If this were the case, it could explain effects of FTR status. A crucial point is that risk may be an overarching confounding factor in many outcomes involving real-world behaviour assumed to be impacted by time preferences. Most real-world intertemporal decisions involve some degree of risk. For instance, someone deciding whether to smoke a cigarette has to balance the present pleasure against future costs as a function of delay and probability, i.e. *when* and *if* she will develop smoking-related health issues. Many of the outcomes predicted by FTR status involve real-world intertemporal decision making involving risk. If strong-FTR speakers construe future outcomes as riskier, effects of FTR status might result from a difference in risk preferences, i.e. $F_{S}(p_{FUT})<F_{W}(p_{FUT})$ (which is the opposite direction to the high-certainty hypothesis we have outlined above). If the obligation to use low-certainty language about the future resulted in such beliefs, strong-FTR speakers would discount *more* not *less* than weak-FTR speakers. This is, in fact, what has repeatedly been found, [7,14–22,30,60–67]. Since we have demonstrated that Chen’s (2013) proposed mechanisms are not supported — at least in English and Dutch— we posit that this “modal” account may be responsible for observed findings.

### Limitations

There are several limitations to the present study which should be discussed. First, in Study 2, the participants were recruited from different platforms (English from Prolific, Dutch from Qualtrics). This was done for practical reasons (the Dutch population on Prolific is limited and due to funding constraints, we were unable to use Qualtrics, which is more costly, for both samples). It is possible differences in recruiting methods drove results. We consider this to be unlikely since we found the same effects in Study 1, which recruited participants from different populations than Study 2 (English from Oxford students and Prolific, and Dutch from the MPI). Replicating findings across different recruitment platforms suggests participant sampling methods were not a determinant of outcomes in either study. Second, data in Study 2 were collected at different times for the different samples (Dutch: 2018; English: 2020). It could be the case that global events drove observed findings (e.g., COVID-19, inflation). We suggest this is unlikely on the grounds that Study 2 replicated Study 1, which recruited both language samples contemporaneously in 2017. Additionally, whilst exhibiting some state-like characteristics, delay discounting has strong trait-like characteristics and appears to be relatively stable across time, context, and outcome [70]. Third, the values in the intertemporal choice task may not be representative of real world situations, and necessarily do not represent a full-spectrum comparison of English and Dutch speakers across all reasonable reward values. This is important since reward value does change discounting function curves [71]. More research would be needed with larger reward values to better understand the range of values for which the findings we report may replicate.

The Dutch sample should be discussed. Dutch speakers may use English frequently (especially the kind of population who participate in online surveys). If speaking a language with obligatory FTR marking affects intertemporal decision making, this could have made Dutch-English bilinguals more “English like” in their discounting behaviour. However, even if this were the case, this suggests the mediation analyses we report are conservative, since the Dutch sample would presumably discount more than the Dutch population. Additionally, Dutch *does* have a future tense (just not an obligatory one). Readers may notice that present:future tense ratios were less stable study to study, and exhibited higher variance, in Dutch than English, Table 1. This observation may give rise to reasonable concerns regarding Dutch’s status as a weak-FTR language. However, we point out that greater variance and less stability is exactly what you would expect in speakers of a language with a more weakly gramaticised future, where utterances are guided by idiosyncratic differences between speakers rather than strong grammatical constraints. Nonetheless, Dutch does have a future tense, which may make it difficult to extrapolate from Dutch to languages (like Finnish or Chinese) which do not. Similarly, English does not have an bound morphological future tense (like French or Spanish), which may make it difficult to extrapolate from English to other strong-FTR languages. In fact, both Dutch and English are closely related Western Germanic languages in which the future tense comprises part of a modal verb system. Our conclusion that the future tense may encode the modal notion of high-certainty should be seen in this context. More research is needed to understand how general the effects we report may be. However, we suggest building from our approach will be useful for future research. We have closely investigated the mechanisms that underpin relationships between FTR marking and discounting in a small sample of 2 languages. This has afforded a more granular understanding of the FTR systems in question, but necessarily sacrifices generalisability. However, we suggest that more close examinations of language-wise contrasts in FTR marking and intertemporal choice in small samples of languages will better advance future research than broad cross-linguistic correlational research which necessarily paints cross-linguistic differences in broad strokes.

### Implications for the linguistic relativity hypothesis

The present results support the linguistic relativity hypothesis. We found that grammatical differences between languages caused differences in usage between speaking populations (English speakers used more future tense constructions). This in turn was associated with a difference in non-linguistic cognition: decreased discounting in English speakers relative to Dutch. A difference between populations does not necessarily constitute evidence for relativity, as factors other than language might be responsible. However, the mediation analyses were able to statistically quantify the relationship between certain grammatical constructions (the future tense) and discounting. This suggests language is causally implicated in observed differences in discounting, supporting the linguistic relativity hypothesis.

## Conclusion

Psychological discounting processes are an important determinant of a wide range of behaviours, including health outcomes [72], drug use [73], pathological gambling [74], and investment in savings [75]. If the correlation between FTR grammaticisation and intertemporal preferences is not spurious, the breadth of reported findings attest to the impact cross-linguistic differences may have on the discounting processes which underpin these behaviours. Understanding the causal mechanisms involved will be critical for research to generate usable policy applications or therapeutic interventions. Our research indicates that the causal model espoused in much of this research should be reconsidered. We consistently found that use of the future tense was associated with less not more discounting. We therefore conclude that obligatory use of low-certainty modal FTR terms is more likely to be responsible for observed linguistically-driven differences in behaviours involving intertemporal decision making.

## Supporting information

SI 1 Text Supplementary information for “Language and economic behaviour: future tense use causes less not more temporal discounting” (PDF)
