# Supplementary material for: Language and economic behaviour: Future tense use causes less not more temporal discounting
Source: PLoS One. 2025 May 27;20(5):e0317422. doi: 10.1371/journal.pone.0317422 (PMC12112090; doi:10.1371/journal.pone.0317422)
Supplement: SI 1 Text — (PDF) [file pone.0317422.s001.pdf]

Supplementary Materials for:

Language and economic behaviour: Future tense use causes less not more temporal discounting

Cole Robertson*

Radboud University

Seán G. Roberts

Cardiff University

Asifa Majid

University of York

Robin I. M. Dunbar

University of Oxford

Author Note

*corresponding author: The Center for Language Studies, Radboud University, PO Box 9103 6500 HD, Nijmegen, cole@texture.ai

Cross-study analyses

In this section, we present results which are pertinent to Studies 1 and 2. Following this, we present analyses which pertain to each study individually.

FTR-classifier accuracy

See *Table 1* for accuracy metrics for the predictions of the FTR classifier against human raters. Data are drawn from Studies 1 and 2.

FTR elicitation task factor balance

Balance of FTR mode, temporal distance, and probability condition in the FTR elicitation task across experiments are given in *Tables 2–4.* Tables present the counts of unique items per cross-tabulated factors of the FTR elicitation task, for each of Study 1 and 2.

Demographic differences and descriptions

Demographics are reported in *Fig. 1.* In no case did adding any of these variables substantively change parameters of interest, or significantly improve model fit, $p < .05$. They were therefore disregarded.

Study 1

Participant exclusions

Data for Study 1 was collected between January and February, 2017. One participant was excluded from the English condition because his/her answers to the FTR elicitation task made it evident that he/she did not provide a good-faith response.

Selecting items to be used in the FTR-elicitation task

To chose the items which we would use in Study 1, we conducted a pilot where we collected English and Dutch FTR usage data using the full (48 item) FTR elicitation task. Data were collected between July and October, 2016, from the MPI participant

panel (Dutch and German), and Oxford University students (English). Final $N = 64$ ($n = 45$ English and $n = 19$ Dutch). Ethical approval for the study was granted by the University of Oxford internal review board, ref. no. R39324/RE001, and by the MPI for the data collection that occurred there. All participants were remunerated. To choose the questions to be used in Study 1, we regressed future tense use over an intercept, which we allowed to vary by item, and participant. We also allowed slopes for language to vary by item so that random slopes would be estimated for both English and Dutch:

$$log_e(\frac{\pi_{ijk}}{1-\pi_{ijk}}) = \beta_0 + (\beta_1 + s_k)LANG_{ijk} + p_j + q_k \tag{1}$$

where π_{ijk} is the probability that $y_{FUT} = 1$ for observation i, participant p_j, question q_k, and language. Random intercepts and slopes, p_j, q_k, s_j, are assumed to be drawn from the normal distributions with means of 0 and standard deviations observed in the sample, i.e. $p, q, s \sim N(0, \sigma^2_{p,q,s})$. We then sorted in ascending order by the absolute value of the random slope term, s_k, for each language. The allowed us to select the top $n = 20$ items for each language for which the item slope was closest to 0, i.e. the items which had the least constrained contexts in terms of use of the future tense. To create a matched sample of items, we took the union of the results for English and Dutch. This resulted in $N = 29$ unique items.

The item numbers for the resultant set of items are: 1001, 1002, 1003, 1005, 1006, 1007, 1009, 1010, 1011, 1012, 1013, 1015, 1016, 1018, 1019, 1021, 1025, 1027, 1028, 1032, 1033, 1034, 1035, 1037, 1039, 1041, 1045, 1046, and 1048, see *Table 5*.

Table 5 presents English items. To see Dutch translations, go to the FTR-elicitation task Github repository at: `https://github.com/cbjrobertson/ftr_questionnaire_master/tree/master/ftr_questionnaire/versions_1-2`.

Item exclusions

Of the 29 items in the FTR elicitation task, one needed to be removed from analysis because because it was later realised that the prompt included common modal or tense words (e.g. *think*, *expect*, *go*) which prevented the FTR classifier from

accurately identifying whether participants had used a word themselves, or whether they had simply copied the prompt. Additionally, one question needed to be removed because inspection revealed that participants consistently interpreted the context as referring to present time, and one question needed to be removed because it was in both these categories. A total of three questions were therefore removed, so analysis was conducted on data from 26 of the 29 items participants were given.

Study 2

Participants exclusions

Data for Study 2 were collected between October and November, 2018 (Dutch), and in May, 2020 (English). In the Dutch condition, of $n = 323$ complete responses, 16 were excluded because either the responses contained too much missing data (10 responses) or inspection revealed the answers were given in bad faith (6 responses). Of the $n = 301$ complete responses which passed attention checks in English, none were excluded. Final $N = 608$ ($n = 301$ in English, and $n = 307$ in Dutch).

Item exclusions

The final set of FTR elicitation task questions was identical to Study 1, though an additional two items had to be excluded because they accidentally used an absolute time frame which at the time the experiment was conducted was in the present/past ("by 2018"). This means the final number of elicitation questions analysed is 24 items.

Power analysis

In order to arrive at an optimal number of participants for Study 2, we conducted a power analysis using the *R* package *simr* (Green & MacLeod, 2016). The power of an hypothesis test can be defined as the "probability that a test will reject the null hypothesis, assuming that the null hypothesis is false" (Green & MacLeod, 2016, p. 493). In other words, it is the probability that a hypothesis test will detect a true effect. A standard acceptability rate is 80% (Green & MacLeod, 2016). The *simr* package

(Green & MacLeod, 2016) calculates power for generalised mixed models estimated with the *lme4* package (Bates, Mächler, Bolker, & Walker, 2015), so it was well-suited to our needs. Power calculations in *simr* are based on Monte Carlo simulations which allow researchers to simulate power under various hypothetical scenarios. In this way, results can inform trade-offs between sample size and power (Green & MacLeod, 2016). Basically, researchers can specify a mixed model in *lme4*, and then use *simr* to understand the power of the model given previous data, and across hypothetical values for fixed effects and sample sizes.

Our statistical approach at the time we were planning Study 2 was different from the analyses we present in the paper (we thank our reviewers for pointing us towards better statistical approaches). This means our power analysis was based on a different model structure than we report in the main text. However, since it informed our decisions about sample size, it is reported here for transparency. We were interested in the impact of future tense use on time preferences. At the time, we were using mixed logistic regressions to regress raw Inter Temporal Choice (ITC) values (SSR = 0; LLR = 1) over participant-level mean future tense use, and allowing intercepts to vary by participant, SSR amount, and LLR delay:

$$log_e(\frac{\pi_{ijkl}}{1-\pi_{ijkl}}) = \beta_0 + \beta_1 FUT_i + p_j + d_k + a_l \quad (2)$$

where π_{ijkl} is the probability that $y_{ITC} = 1$ for observation i, participant p_j, delay d_j, amount a_l. Random intercepts, $p_j,\ d_k,\ a_l$, are assumed to be drawn from the normal distributions with means of 0 and standard deviations drawn from the sample, i.e. $p, d, a \sim N(0, \sigma^2_{p,d,a})$. When we estimated this model using the data from Study 1, the effect of future tense was $\beta_1 FUT = 0.26$. In our simulations, we therefore used 0.25 as our lower bound, and simulated potentially larger effects between $[0.25, 0.4, 0.75, 0.9]$. We then calculated power for a sample of $N = 0$–$N = 300$ participants (the largest sample our budget allowed) for each hypothetical $\beta_1 FUT$.

Results are reported in *Fig. 3.* They are not particularly promising. They suggest that even with $N = 300$ participants, Study 2 may be underpowered for $\beta_1 FUT = 0.25$

and $\beta_1 FUT = 0.4$. As $\beta_1 FUT$ increases, so does power: for $\beta_1 FUT = 0.75$, approximately $N = 175$ participants are necessary to reach 80% power; for $\beta_1 FUT = 0.9$, this drops to $n = 120$. Since our observed $\beta_1 FUT$ was 0.25, a large sample was appropriate and decided to collect data from $n = 300$ participants in each language.

Delay and amount adjustment procedure

The amounts and delays in Study 1 appeared to be too distant from relevant decision boundary criteria. For instance, 82.64% of Dutch participants choice the larger-later reward 100% of the time for one-day delays. To choose the amounts and delays for Study 2, we used binary logistic regression to regress raw Inter Temporal Choices (ITCs) (SSR = 0; LLR = 1) over our language dummy, future tense use, LLR delay, and SSR amounts. We included all two- and three-way interactions, and allowed intercepts to randomly vary by participant:

$$\begin{aligned} log_e(\frac{\pi_{ij}}{1-\pi_{ij}}) = \beta_0 &+ \beta_1 LANG_i + \beta_2 FUT_i + \beta_4 DEL_i + \beta_4 AMT_i + \\ &\beta_5 LANG_i : FUT_i + \\ &\beta_6 LANG_i : DEL_i + \\ &\beta_7 LANG_i : AMT_i + \\ &\beta_8 FUT_i : DEL_i + \\ &\beta_9 FUT_i : AMT_i + \\ &\beta_{10} AMT_i : DEL_i + \\ &\beta_{11} LANG_i : FUT_i : DEL_i + \\ &\beta_{12} LANG_i : FUT_i : AMT_i + \\ &\beta_{13} LANG_i : DEL_i : AMT_i + \\ &\beta_{14} FUT_i : DEL_i : AMT_i + \\ &+ p_j \end{aligned} \tag{3}$$

where π_{ij} is the probability that $y_{ITC} = 1$ for observation i and participant p_j. The random intercepts, p_j, are assumed to be drawn from the normal distributions with means of 0 and standard deviations drawn from the sample, i.e. $p \sim N(0, \sigma_p^2)$. Amount was operationalised as the raw SSR amount values; delay was operationalised as the natural log of the number of days. This allowed us to estimate the probability of choosing the LLR over future tense use across the LLR delays and SSR amounts under the study. Data were the data from Study 1. This analysis indicated amounts approaching the larger-later reward (i.e. $> £7$) and with delays between 2 and 6 months had the strongest correlations with future tense use, e.g. steeper slopes can be observed over future tense use in *Fig. 2*. We therefore adjusted the amounts and delays in Study 2 to approximate these ranges.

Tables

Table 1

FTR classifier performance metrics by usage variable

usage variable	accuracy	precision	recall	f1
future	0.98	0.99	0.99	0.99
present	0.96	0.97	0.97	0.97

By any metric, the performance of the FTR classifier is good. It should be noted that it is highly likely the FTR classifier is overfit to these data, though this is not a problem: the FTR classifier is designed to be used on data generated by the FTR questionnaire, so does not need to generalise beyond this constrained domain. Accuracy is defined as $a = (tp + tn)/(tp + fp + fn + tn)$ where tp is the number of true positives, tn is the number of true negatives, fp is the number of false positives and fn is the number of false negatives. Accuracy captures the classifier's performance without prioritising either positive or negative examples. Precision is $p = tp/(tp + fp)$; it captures the model's likelihood of being correct if it makes a positive prediction and is therefore sensitive to the model's type I error rate. Conversely, denominator in recall is the false negatives, $r = tp/(tp + fn)$; it therefore captures whether the model tends to miss true examples, and is sensitive to the model's type II error rate. F1 is the harmonic mean of recall and precision, $F1 = (2rp)/(r + p)$, and attempts to balance the two.

Table 2

FTR task cross-tabs: FTR mode x certainty cond.

study	FTR mode	certainty cond.	Dutch	English
Study 1	intention	certain	2	2
	intention	neutral	3	3
	prediction	certain	3	3
	prediction	neutral	11	11
	prediction	uncertain	6	6
	scheduling	neutral	1	1
Study 2	intention	certain	2	2
	intention	neutral	3	3
	prediction	certain	2	2
	prediction	neutral	11	11
	prediction	uncertain	5	5
	scheduling	neutral	1	1

Table 3

FTR task cross-tabs: FTR-mode x temporal distance

study	FTR mode	temporal distance	Dutch	English
Study 1	intention	one week	3	3
	intention	six months	1	1
	intention	tomorrow	1	1
	prediction	indeterminate	2	2
	prediction	one month	1	1
	prediction	one year	6	6
	prediction	ongoing prediction	2	2
	prediction	six months	2	2
	prediction	ten years	2	2
	prediction	today	1	1
	prediction	tomorrow	1	1
	prediction	twenty five plus	2	2
	prediction	two years	1	1
	scheduling	one year	1	1
Study 2	intention	one week	3	3
	intention	six months	1	1
	intention	tomorrow	1	1
	prediction	indeterminate	2	2
	prediction	one month	1	1
	prediction	one year	4	4
	prediction	ongoing prediction	2	2
	prediction	six months	2	2
	prediction	ten years	2	2
	prediction	today	1	1
	prediction	tomorrow	1	1
	prediction	twenty five plus	2	2
	prediction	two years	1	1
	scheduling	one year	1	1

Table 4

FTR task cross-tabs: certainty cond. x temporal distance

study	certainty cond.	temporal distance	Dutch	English
Study 1	certain	one week	1	1
	certain	one year	2	2
	certain	six months	1	1
	certain	tomorrow	1	1
	neutral	indeterminate	1	1
	neutral	one month	1	1
	neutral	one week	2	2
	neutral	one year	2	2
	neutral	ongoing prediction	1	1
	neutral	six months	2	2
	neutral	ten years	1	1
	neutral	today	1	1
	neutral	tomorrow	1	1
	neutral	twenty five plus	2	2
	neutral	two years	1	1
	uncertain	indeterminate	1	1
	uncertain	one year	3	3
	uncertain	ongoing prediction	1	1
	uncertain	ten years	1	1
Study 2	certain	one week	1	1
	certain	one year	1	1
	certain	six months	1	1
	certain	tomorrow	1	1
	neutral	indeterminate	1	1
	neutral	one month	1	1
	neutral	one week	2	2
	neutral	one year	2	2
	neutral	ongoing prediction	1	1
	neutral	six months	2	2
	neutral	ten years	1	1
	neutral	today	1	1
	neutral	tomorrow	1	1
	neutral	twenty five plus	2	2
	neutral	two years	1	1
	uncertain	indeterminate	1	1
	uncertain	one year	2	2
	uncertain	ongoing prediction	1	1
	uncertain	ten years	1	1

Table 5

FTR-elicitation questionnaire for Studies 1 and 2 (English)

question	question number	FTR-mode	temporal distance	modality condition
[Q: Do you think your dad will go to sleep?] A: Yes, he {BE} tired.	1001	prediction	today	neutral
[Q: What your brother {DO} if you don't go to see him today, do you think?] A: He {CALL} me (on the phone).	1002	prediction	today	neutral
[It's no use trying to swim in the lake tomorrow...] ...The water {BE} cold (then).	1003	prediction	tomorrow	neutral
The sun {RISE} at six O'clock tomorrow.	1004	scheduling	tomorrow	neutral
[The boy's father sent him a sum of money some days ago and it will arrive next week.] (The boy {GET} the money next week. When {GET} it...) ...he {BUY} a present for the girl.	1005	intention	one week	neutral
[My brother {SAY} yesterday...] ...that he {COME} here next week.	1006	intention	one week	neutral
[Don't invest in derivatives. The market is fraudulent...] ...It {CRASH} within a month.	1007	prediction	one month	neutral

Continued on next page...

Table 5 – *FTR-elicitation questionnaire for Studies 1 and 2 (English) continued*

question	question number	FTR-mode	temporal distance	modality condition
[Father to son: If you {PUT} your allowance in a savings account...] ...next month it {BE} worth more.	1008	prediction	one month	neutral
[Talking about a third person's plan for the summer (it is presently December):] (In the summer) he {TRAVEL} to Morocco.	1009	intention	six months	neutral
[Talking about the summer's upcoming weather (it is presently December):] (There {BE} a warm current coming from the tropics this year...) ...It {MAKE} this summer hot and rainy.	1010	prediction	six months	neutral
[If I keep eating pizza every day...] ...I {BE} fat by the end of the year.	1011	prediction	one year	neutral
[Q: You {STAY} here, next year?] A: No, I {LIVE} in San Francisco (next year).	1012	intention	one year	neutral
[Don't bother investing in real estate...] ...the housing market {CRASH} in the next 2 years.	1013	prediction	two years	neutral
[Don't buy those shoes...] ...they {WEAR OUT} in a couple of years.	1014	prediction	two years	neutral

Continued on next page...

Table 5 – *FTR-elicitation questionnaire for Studies 1 and 2 (English) continued*

question	question number	FTR-mode	temporal distance	modality condition
[Q: Are you {SELL} both your houses? A: We {WAIT}. Maybe in another ten years. Real estate always goes up in value...] ...By then, they {BE} much more valuable.	1015	prediction	ten years	neutral
[Q: What {BE} your ten year projection for Coca-Cola stock? A: I {THINK} they have good fundamentals. I {THINK} in ten years...] ...they {GAIN} 10% or even 20%.	1016	prediction	ten years	neutral
[Q: Do you {WANT} a cigarette? A: No, I don't smoke...] ...If you smoke you {GET} cancer (when you are old).	1017	prediction	twenty five plus	neutral
[Q: You {BE} saving for retirement? A: Yes, I {PUT} away č100 every month. By the time I {RETIRE}...] ...it {BE} worth č50,000, with interest.	1018	prediction	twenty five plus	neutral
[You should {PUT} money into a retirement savings plan; when you {BE} older...] ...you {THANK} yourself.	1019	prediction	twenty five plus	neutral
[If you {PUT} a stone into this bag...] ...the bag {BREAK}.	1020	prediction	ongoing prediction	neutral

Continued on next page...

Table 5 – *FTR-elicitation questionnaire for Studies 1 and 2 (English) continued*

question	question number	FTR-mode	temporal distance	modality condition
[A: I have a headache. B: Take this medicine...] ...It {MAKE} you feel better.	1021	prediction	ongoing prediction	neutral
[Q: What {HAPPEN} if you eat this mushroom?] A: You {DIE}.	1022	prediction	ongoing prediction	neutral
[A sexual health instructor speaking to class: If you {HAVE} sex without protection...] ...you {HAVE} a baby.	1023	prediction	ongoing prediction	neutral
[Q: What you DO this evening? A: I'm not sure...] ...I {GO} out to dinner with a friend (but I'm waiting for her to call).	1024	intention	today	low certainty
[Talking about a third person's plans for the evening: I {BE} not sure...] ...He {WORK} in the garden. (He usually {DO} on Sunday).	1025	prediction	today	low certainty
[Q: What you {DO} tomorrow evening? A: It depends how I feel. There {BE} a film playing that I {WANT} see at 7...] ...I {GO} with Mary.	1026	intention	tomorrow	low certainty

Continued on next page...

Table 5 – *FTR-elicitation questionnaire for Studies 1 and 2 (English) continued*

question	question number	FTR-mode	temporal distance	modality condition
[Q: Are you sure that you want to go? A: I've decided. I {LEAVE} tomorrow...] ...In two days, I {BE} in Australia (whether you {BE} with me or not).	1027	intention	tomorrow	neutral
[Q: When does your mother arrive? A: Ug. In a week. She {INSIST} on {BUY} the kids too much sugar, like always...] ...I {SAY} no this time (definitely).	1028	intention	one week	neutral
[Q: What is the weather forecast for next week?] A: It {RAIN} (at least it says it's 50% likely).	1029	prediction	one week	low certainty
[Q: How do expect the markets {BEHAVE} next month? A: I {THINK}...] ...they {RISE} (but it is uncertain).	1030	prediction	one month	low certainty
[Q: How do expect the markets {BEHAVE} next month? A: Next month, I {EXPECT}...] ...they {RISE} (but I am not sure).	1031	prediction	one month	low certainty
[Q: How do expect the markets {BEHAVE} in the next 6 months?] A: They {RISE} (I am certain of it!).	1032	prediction	six months	neutral

Continued on next page...

Table 5 – *FTR-elicitation questionnaire for Studies 1 and 2 (English) continued*

question	question number	FTR-mode	temporal distance	modality condition
[Don't bother investing in real estate...] ...next year, the housing market {CRASH} (it is a possibility).	1033	prediction	one year	low certainty
[Don't bother investing in real estate...] ...the housing market {CRASH} (next year, potentially).	1034	prediction	one year	low certainty
[Don't bother investing in real estate...] ...(I'm very sure) the housing market {CRASH} (next year).	1035	prediction	one year	neutral
[Q: How do expect the markets {BEHAVE} next year?] A: They {RISE} (it is likely, in my opinion).	1036	prediction	one year	low certainty
[Don't bother investing in the tech industry...] ...Silicon Valley {CRASH} by 2018 (probably but not certainly).	1037	prediction	two years	low certainty
[Don't bother investing in the tech industry...] ...Silicon Valley {CRASH} by 2018 (it's possible).	1038	prediction	two years	low certainty
[Don't bother investing in the tech industry...] ...Silicon Valley {CRASH} by 2018 (definitely).	1039	prediction	two years	neutral

Continued on next page...

Table 5 – *FTR-elicitation questionnaire for Studies 1 and 2 (English) continued*

question	question number	FTR-mode	temporal distance	modality condition
[Don't bother investing in real estate...] ...the housing market {COLLAPSE} (in the next ten years, probably).	1040	prediction	ten years	low certainty
[Don't bother investing in real estate...] ...the housing market {COLLAPSE} (in the next ten years, it's a potential but not certain).	1041	prediction	ten years	low certainty
[Q: Do you {WANT} a cigarette? A: No, I Don't smoke...] ...If you smoke you {GET} cancer (when you are old, I'm fairly sure).	1042	prediction	twenty five plus	low certainty
[Q: Do you {WANT} a cigarette? A: No, I Don't smoke...] ...If you smoke you {GET} cancer (when you are old, definitely. I'm certain).	1043	prediction	twenty five plus	neutral
[Q: Do you {WANT} a cigarette? A: No, I Don't smoke...] ...If you smoke you {GET} cancer (when you are old, probably).	1044	prediction	twenty five plus	low certainty
[Don't bother investing in real estate...] ...the housing market {CRASH} (it's possible).	1045	prediction	indet.	low certainty

Continued on next page...

Table 5 – *FTR-elicitation questionnaire for Studies 1 and 2 (English) continued*

question	question number	FTR-mode	temporal distance	modality condition
[Q: Do you {WANT} a cigarette? A: No, I Don't smoke. I {BE} afraid...] ...I {GET} cancer.	1046	prediction	indet.	neutral
[The boy thinks he will perhaps get a sum of money: If the boy {GET} the money...] ...he {BUY} a present for the girl.	1047	intention	ongoing prediction	neutral
[Q: Do you {WANT} a cigarette? A: No, I Don't smoke...] ...If you smoke you {GET} cancer (it's possible).	1048	prediction	ongoing prediction	low certainty

Figures

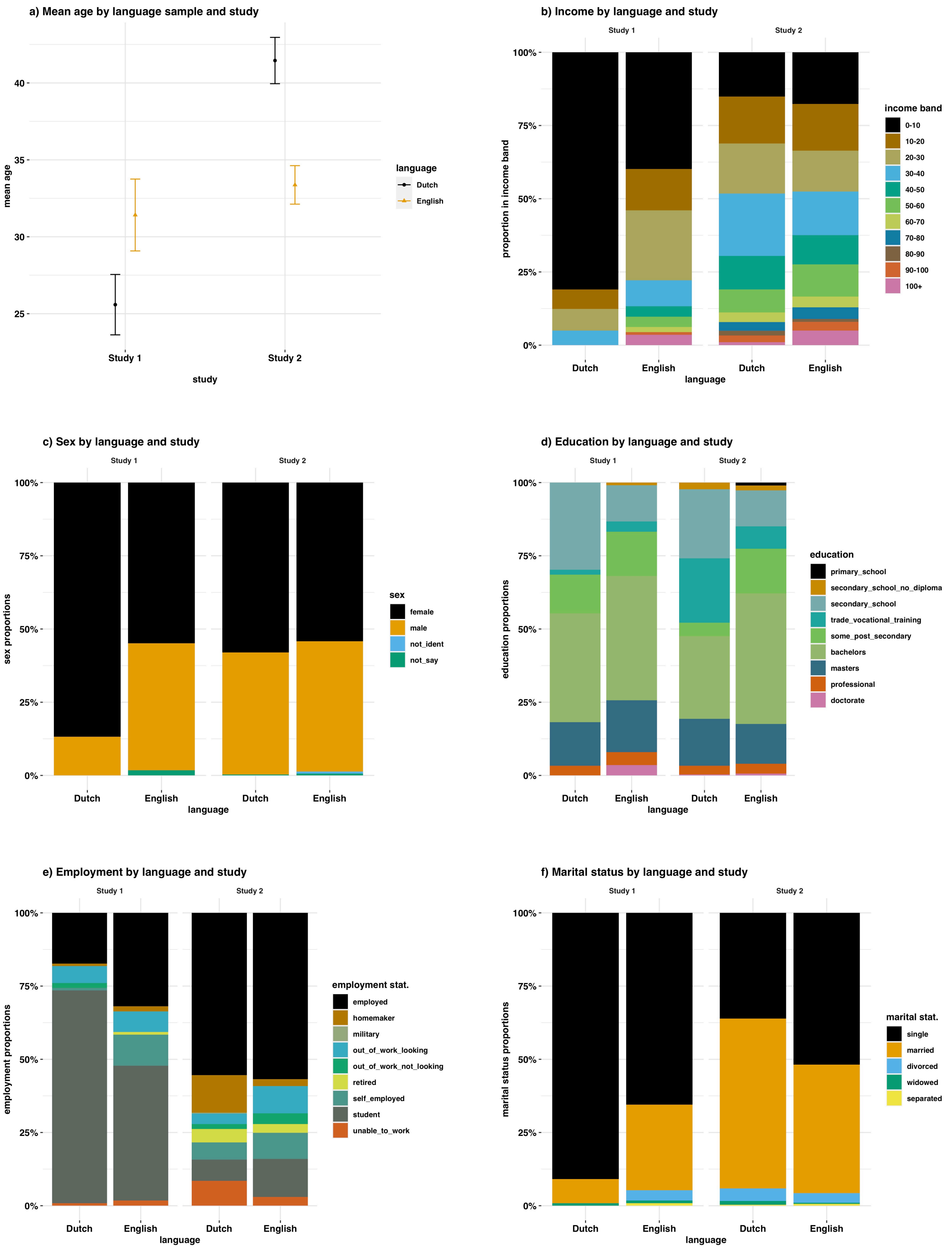

Figure 1. Demographic sample composition by langauge and study. $Mean \pm 1\ 1.96SE$ is reported for sex (a). Other subfigures (b-f) report preportions.

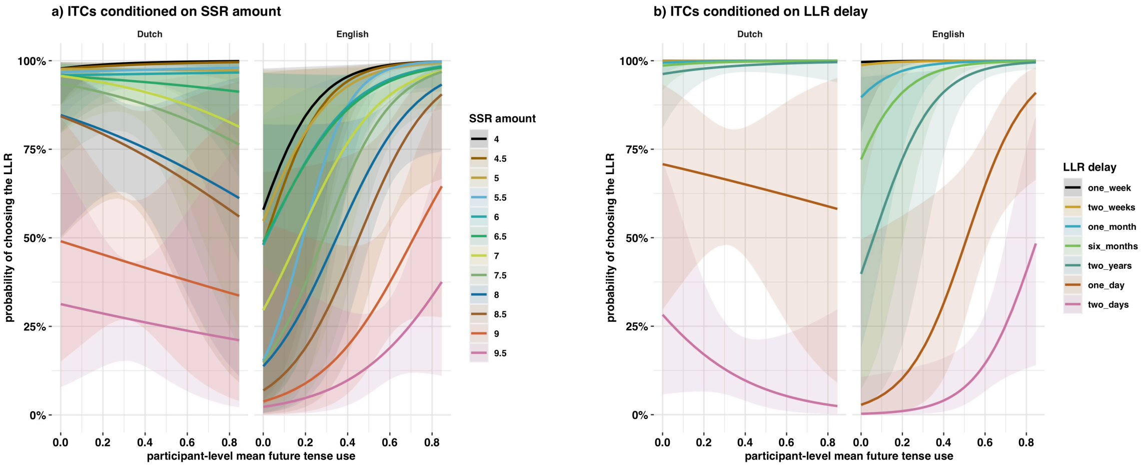

Figure 2. Probability of choosing the LLR over participant-level mean future tense use in Study 1. Probabilities are conditioned on SSR amounts and delays. Plotted values are the marginal effects, ± 1.96 SE (see Lüdecke, 2019). For ease of interpretation, adjectival delays are shown rather than $ln(days)$ in (b).

(a) $\beta FUT = 0.25$

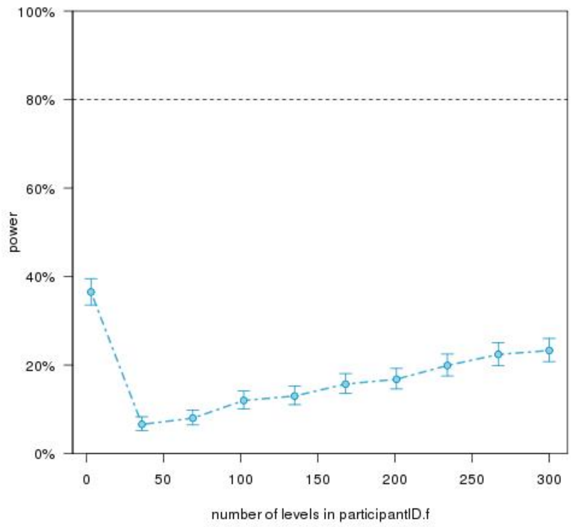

(b) $\beta FUT = 0.4$

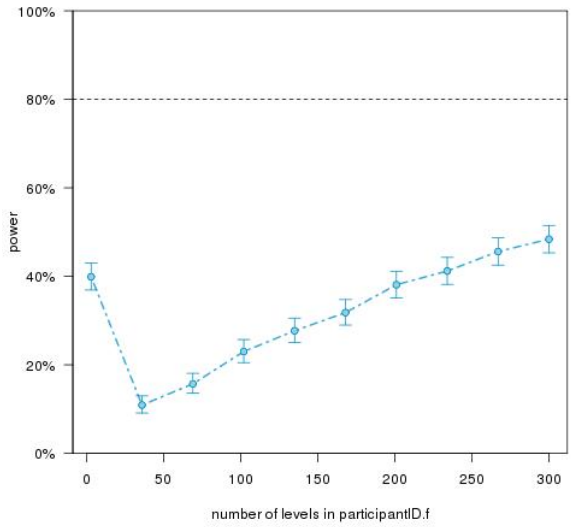

(c) $\beta FUT = 0.75$

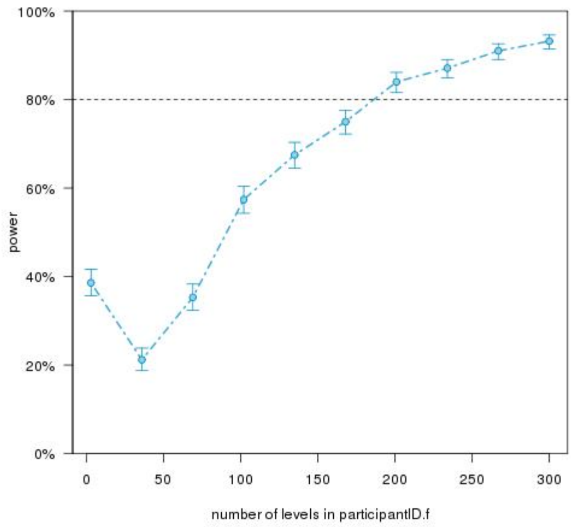

(d) $\beta FUT = 0.9$

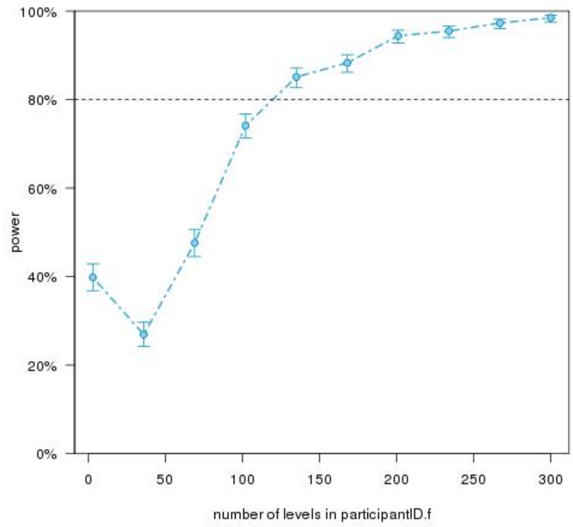

Figure 3. Simulated power under different hypothetical sample and effect sizes, according to our *simr* analysis. The standard cut-off of 80% is depicted (Green & MacLeod, 2016). Simulated power is plotted on the y-axes. Simulated sample size (number of participants) is plotted on the x-axes.

References

Bates, D., Mächler, M., Bolker, B. M., & Walker, S. C. (2015). Fitting linear mixed-effects models using lme4. *Journal of Statistical Software*, *67*(1). doi: 10.18637/jss.v067.i01

Green, P., & MacLeod, C. J. (2016). simr: An *R* package for power analysis of generalised linear mixed models by simulation. *Methods in Ecology and Evolution*, *7*(4), 493–498. doi: 10.1111/2041-210X.12504

Lüdecke, D. (2019). *ggeffects: Marginal effects of regression models.* Retrieved from `https://strengejacke.github.io/ggeffects/articles/ggeffects.html`
